# Antioxidants Present in Reproductive Tract Fluids and Their Relevance for Fertility

**DOI:** 10.3390/antiox10091441

**Published:** 2021-09-09

**Authors:** João C. Ribeiro, Patrícia C. Braga, Ana D. Martins, Branca M. Silva, Marco G. Alves, Pedro F. Oliveira

**Affiliations:** 1 Department of Anatomy, Unit for Multidisciplinary Research in Biomedicine (UMIB), Institute of Biomedical Sciences Abel Salazar (ICBAS), University of Porto, 4050-313 Porto, Portugal; joaopcribeiro95@gmail.com (J.C.R.); patriciacbraga.1096@gmail.com (P.C.B.); alvesmarc@gmail.com (M.G.A.); 2 QOPNA & LAQV, Department of Chemistry, University of Aveiro, 3810-193 Aveiro, Portugal; anacdmartins@gmail.com; 3 CICS, Faculty of Health Sciences, University of Beira Interior, 6201-001 Covilhã, Portugal; branca@fcsaude.ubi.pt

**Keywords:** antioxidants, fertility couple, reactive oxygen species, female reproductive fluid, seminal plasma, sperm parameters

## Abstract

Nowadays, infertility is classified as a disease of the reproductive system. Although it does not compromise the life of the individual, it can have detrimental effects on the physiological and psychological health of the couple. Male fertility evaluation is mainly focused on the analysis of sperm parameters. However, the ejaculated fluid is also composed of seminal plasma, and the study of this fluid can provide crucial information to help in the assessment of male fertility status. Total antioxidant capacity of the seminal plasma has been positively correlated with the fertility of men. Moreover, evidence highlights to a similar importance as that of female reproductive tract fluid antioxidant capabilities and female fertility. Herein, we describe the functions of seminal plasma and female reproductive tract fluids, as well as their main antioxidant components and their relationships with fertility outcomes. Additionally, this review contains the most up to date information regarding the mechanisms of the interaction between the male and the female reproductive fluids and the importance of proper antioxidant capacity for fertilization.

## 1. Introduction

As a reproductive health disease, infertility is defined as an incapacity to generate and maintain a full-term pregnancy after a year of regular sexual intercourse without the use of any contraceptive method [1]. According to the World Health Organization (WHO), around 9% of the couples worldwide present fertility issues [2]. Females or males may have infertility problems. For many years, infertility issues were mainly attributed to women, but nowadays, such problems are attributed to both sexes in almost equal parts [2]. Male and female reproductive tract fluids have a crucial role on fertility. Indeed, the fertility of the couple is dependent on the chemical composition of these fluids. Seminal plasma in men and vaginal, uterine, and oviductal fluid in the female reproductive tract allow the nourishment of gametes and assist their fertilization. Among all the molecules that compose the reproductive fluids, those with antioxidant capabilities have been gaining interest. Antioxidants levels in reproductive fluids have been positively correlated to human fertility, independent of sex.

In this review, we will discuss the composition of antioxidants in the seminal plasma and female reproductive tract fluids and its relationship with the fertility of individuals. Moreover, the most recent information on the role of the reproductive fluids and their antioxidant profile on the processes that allow fertilization and embryo development will be discussed.

## 2. Constitution and Functions of Seminal Plasma

**Figure 1:**
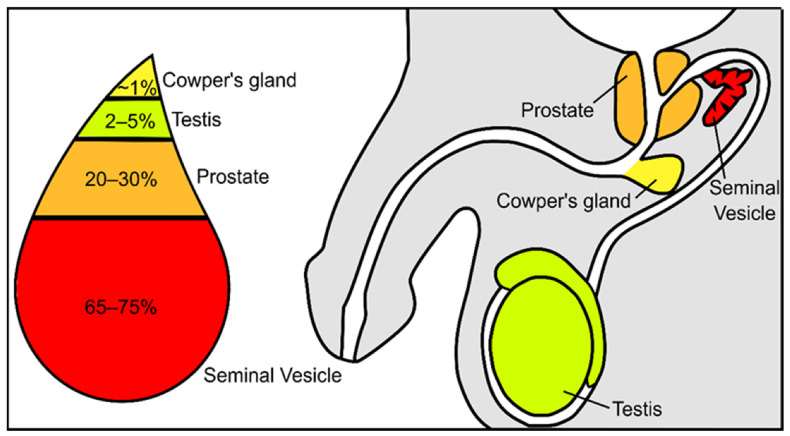
Schematic representation of the contribution from the different urogenital glands to the seminal plasma composition.

Semen constitutes a mixture of cells and liquids in a mixture of 5 to 95%, respectively [3]. The cellular fraction of this fluid constitutes the spermatozoa, epithelial cells, and leucocytes, while the non-cellular liquid fraction of semen is known as seminal plasma. Seminal plasma is a very complex and important fluid in the function of the ejaculate. It is formed by secretions from the rete testis, epididymis, and accessory glands [4,5]. The contribution of each component suffers slight variations: 2–5% are secretions from the testis, 20–30% from the prostate, approximately 1% from bulbourethral and periurethral glands, and the major contribution is 65–75% from the seminal vesicles (Figure 1) [3]. Fructose and other sugars, amino acids, peptides and proteins, lipids, cytokines, microRNA, and inorganic ions are its main constituents.

At first look, the role of seminal plasma seems to be related to the survival and transport of spermatozoa; however, this has been devaluated by the assisted reproductive technologies [5]. In fact, washed spermatozoa produce viable embryos. On the other hand, a lower fertility is observed when seminal plasma is diluted or removed in livestock species [6]. Although contradictory information can be found in the literature, is not possible to deny that seminal plasma must have a role in sperm function and in events in the male and female reproductive tracts, such as fertilization and embryo development [7]. Thus, it is worth studying the seminal plasma of men with fertility problems. The measurement of zinc, fructose, and neutral α-glucosidase content in seminal plasma are optional assays suggested by the WHO laboratory manual for the examination and processing of human semen [8]. Zinc has a protective effect on spermatozoa from bacteria and is important in testicular development and spermatogenesis [9,10,11]. In seminal plasma, fructose is mainly produced by the seminal vesicles [12] and has a role in spermatozoa metabolism [13] as a major energy source [14] for motility [13]. The determination of seminal fructose is useful in cases of inflammation of accessory glands and in cases of obstructive azoospermia. The content of fructose is low or undetectable when ejaculatory ducts are obstructed [15] or a congenital vas deferens-seminal vesicle developmental defect is present [16,17]. The determination of fructose concentration, as auxiliary diagnosis, is also useful in cases of retrograde ejaculation, when an abnormal concentration of this sugar is detected in urine after ejaculation [15]. The α-glucosidase is a marker for the epididymal function [18,19]; however, the measurement of α-glucosidase is not widely implemented and used [20]. Alterations in α-glucosidase are associated with a defective process of spermatozoa maturation in epididymis or inflammation in this structure [21,22].

The roles of seminal plasma, from the epididymis to fertilization, were studied in several species, although the available data highlights contradictory conclusions. Seminal plasma functions include: effects on sperm motility, mostly related to proteins found in this fluid [23,24,25]; providing nutrients and osmotic homeostasis for spermatozoa survival [26,27,28,29]; effects on the capacitation process and also on the avoidance of premature activation of capacitation [30,31,32]; an active role in the inflammatory response by the female reproductive tract [33,34,35]; the modulation of sperm-egg interactions; and effects on fertilization [36,37,38]. The importance of seminal fluid in fertilization events was investigated in studies where seminal plasma components were added to thawed sperm. It was reported that seminal plasma acts as a defense mechanism for spermatozoa from cells from the immune system [39]. Supplementation of cryopreservation medium with seminal plasma was proved to improve motility, membrane integrity, and in vitro fertilization (IVF) outcomes [40]. Similarly, it was described that seminal plasma added to post-thawed sperm medium has beneficial effects on sperm motility and membrane integrity [41,42]. This could be due to the known antioxidant capabilities of seminal plasma, which protects the sperm cells from oxidative damage. The antioxidant capabilities of seminal plasma are due to multiple nutrients of different sources on the male reproductive tract (Table 1). These help in the control of excessive reactive oxygen species (ROS) production. Dysregulation of antioxidant secretion can lead to oxidative stress in the semen, which has been regarded as one of the main causes of male infertility in recent decades [43].

## 3. Seminal Plasma Antioxidant Capabilities and Relation to Male Fertility Outcomes

Basic semen analysis is based on the macroscopic and microscopic observation of the ejaculated and is, nowadays, considered the gold standard for male fertility evaluation based on the limits established by the WHO [8,49]. It is a reality that, currently, andrologists and specialists in the field recognize and agree that it does not provide sufficient information for the evaluation of fertility in some cases [24,50,51,52]. It is important to consider the limitation of this technique related to the variability [49], and the information provided by the basic semen analysis needs to be analyzed and conjugated with historic physical examinations and other clinical information [49].

Although spermatozoa are the main agents of male fertility, the WHO suggests some optional biochemical assays on seminal plasma to assess male fertility status [8]. Seminal plasma is the body fluid with a higher concentration of antioxidants [5]. This highlights the importance of the redox balance for sperm cells. A high concentration of seminal plasma antioxidants is necessary due to the low antioxidant defenses present in the limited sperm cytoplasm, which makes sperm cells particularly vulnerable to oxidative damage. Seminal plasma oxidative stress can easily spiral out of control without proper antioxidant capabilities. One of the main sources of seminal plasma ROS are the immature and abnormal sperm cells that have dysregulated mitochondrial function, which results in enhanced ROS production and diffusion [53]. Peroxidase positive leukocytes are also a major ROS producer even in the seminal plasma of healthy men [54]. Normally, in healthy individuals, these ROS do not pose a threat to the sperm cells. In fact, these ROS sources, together with the endogenous ROS production from each sperm cell, are important for fertility since they assist in the process of sperm capacitation. This process is characterized by higher sperm membrane fluidity, mitochondrial function, and culminates in the process of hyperactivation and acrosome reaction. All these events are crucial for natural fertilization; thus, ROS are also essential for fertility. However, excessive ROS can impair male fertility. Diseases and external factors can add to the ROS already present in seminal plasma creating a redox imbalance that ultimately leads to oxidative stress. Oxidative stress has a profound effect on sperm function. Sperm cells incubation with supra-physiologic ROS levels induces impairment of sperm function and inhibition of capacitation [55,56]. The main deleterious effects that oxidative stress has on spermatozoa morphology are considered to be DNA fragmentation, lipid peroxidation and consequent decrease in membrane integrity, and decreased ATP production and consequent decreased motility [57]. Resulting from the sum of all these damages caused by oxidative stress, sperm cells usually suffer apoptosis or lose their motility. Moreover, even after fertilization, embryos from men with high oxidative stress levels on seminal fluid have a higher probability of resulting in miscarriage, probably due to DNA damage and protein oxidation [58,59]. Thus, it is clear the association between seminal oxidative stress and male infertility, which highlighted the relevance of the study of antioxidants and ROS on seminal plasma in understanding male fertility outcomes and the discovery of potential biomarkers for seminal dysfunction.

**Figure 2:**
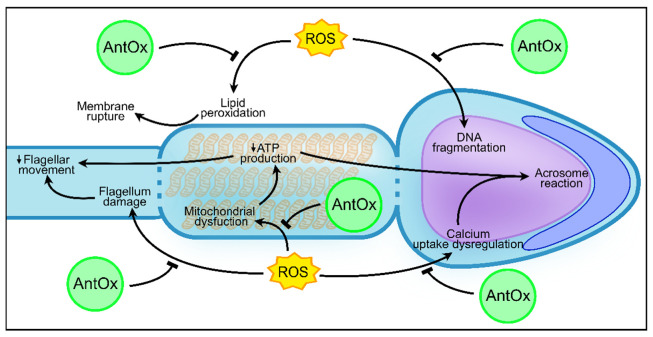
Protective role of antioxidants against oxidative damage in sperm in contact with reactive oxygen species. AntOx—Antioxidants; ROS—Reactive oxygen species; ATP—Adenosine triphosphate.

The total antioxidant capacity of seminal plasma is a very common way of presenting the antioxidant capabilities of the seminal plasma. This term is used to evaluate the total ability of a fluid to scavenge free radicals in solution. In general, fertile men have seminal plasma with a higher total antioxidant capacity than that of infertile patients [60]. Taking this into consideration, different authors have tried to find the correlation between the presence of antioxidants and the fertility outcome. Khosrowbeygi and collaborators developed a study where the total antioxidant capacity of seminal plasma of healthy donors was compared to the one measured in patients with asthenozoospermia, asthenoteratozoospermia, and oligoasthenoteratozoospermia [61]. The levels of total antioxidant capacity were found to be decreased in these patients compared to those of the healthy donors. In a subsequent study developed with 150 male seminal plasma samples, a similar result was found [62]. Normozoospermic presented increased total antioxidant capacity on seminal plasma in relation to men with abnormal sperm parameters. Both these studies highlighted that the total antioxidant capacity of seminal plasma could be an important factor in the maintenance of sperm motility, morphology, and concentration [61,62]. So far, correlation between the total antioxidant capacity and if the individuals have low sperm motility (asthenozoospermia), concentration (oligozoospermia), or normal morphology (teratozoospermia) is yet to be presented. Nevertheless, it must be highlighted that total antioxidant capacity only determines one side of the redox equation. Other methodologies were developed where all the sides of the redox equation were taken into consideration. Recently, oxidation–reduction potential has been referenced as a way of measuring imbalances between ROS present in a solution with the antioxidant capacity of such solution [63]. Agarwal and co-workers showed that non-normozoospermic men had a higher oxidation–reduction potential than did normozoospermic men. Thus, non-normozoospermic men are more prone to have enhanced ROS production or diminished antioxidant capability than normozoospermic men [64]. This makes it clear that either increased ROS levels or low total antioxidant capacity of seminal plasma are major biomarkers for impaired fertility. In fact, 30–80% of infertility cases are due to the presence of one of those factors [43]. However, the relation between the values of oxidation–reduction potential or total antioxidant capabilities of seminal plasma and the motility, concentration, and morphology of the sperm cells is still to be found. One study tried to relate the amount of DNA fragmentation in spermatozoa with the impairment of sperm parameters seen in that individual [65]. The results showed that oligozoospermic men had the most DNA fragmentation when compared to asthenozoospermic, oligoasthenozoospermic, and normozoospermic men. Oligozoospermia is characterized by the low concentration of sperm in the ejaculate. This can be explained by the increased number of apoptotic sperm cells due to oxidative stress in oligozoospermic men and to high oxidation–reduction potential in the seminal plasma [66]. Oxidative damage to DNA, key metabolic proteins, and plasmatic membrane are known triggers of apoptosis [67,68] that is upregulated in oligozoospermic men. This could make one assume that oligozoospermic men are the ones with more oxidative stress in the semen. Additionally, a meta-analysis performed by Buhling and collaborators described that the supplementation of selenium and co-enzyme Q10 (two important known antioxidants) showed to have more positive effects on asthenozoospermic men than in oligozoospermic men [69]. This highlights that motility can be one of the sperm functions most affected by oxidative stress. Sperm from asthenozoospermic men also present high levels of apoptotic sperm cells in comparison to fertile donors [70]. Moreover, in the sperm of these patients, many proteins with roles on ATP production, calcium intake, and flagellum structure, which are intimately involved in sperm motility, were found to have their expression and activity downregulated due to oxidative damage [59,68,71,72] (Figure 2). These facts clearly suggest the possible implication that oxidative damage has on male fertility. It is also worth mentioning that conditions such as teratozoospermia that are characterized by an elevated number of deformed sperm cells in the seminal fluid and non-obstructive azoospermia (which is the total absence of sperm cells in the ejaculate) are commonly related to impaired spermatogenesis [73]. Thus, this is upstream of the formation of the seminal plasma. However, diseases such as testicular infection or cancer can increase ROS production in the area, implementing oxidative stress that can impair spermatogenesis and possibly be in the origin of some individuals with teratozoospermia and non-obstructive azoospermia [74].

Another question worth asking is which antioxidants are of greater importance and which are found to be less represented in males with impaired fertility. As discussed, seminal plasma is a complex fluid, and in its composition are present multiple antioxidants that, herein, will be divided into protein and non-protein. In the next topics, it will be discussed the importance of the abundancy of non-protein antioxidants and the expression and activity of protein antioxidants on seminal plasma for male fertility outcomes (summarized in Table 1).

### 3.1. Non-Protein Antioxidants in Seminal Plasma and Male Fertility

As early as 1995, a study performed by Lewis and collaborators described that the concentration of non-protein antioxidants was found to be different between fertile and infertile men. The difference was greater between fertile normozoospermic men and asthenozoospermic men, which lead the authors to hypothesize that the concentration of these specific groups of antioxidants was related to sperm motility. A diminished concentration of non-protein antioxidants could lead to increased ROS in seminal plasma. Hence, sperm cells would be more vulnerable to oxidative damage [75]. Among all non-protein antioxidants present in semen, vitamin C (l-ascorbic acid) is regarded as the one with the strongest antioxidant capability, since it is one of the most prevalent in the seminal plasma [76]. Interestingly, asthenozoospermic men with seminal plasma with high ROS content have a decrease in vitamin C seminal content when compared to fertile donors [76]. In another study, normozoospermic men also presented a higher seminal content of vitamin C than oligozoospermic and asthenozoospermic men [77]. These two later conditions presented similar vitamin C seminal content, thus decreased sperm motility and concentration in semen are associated with decreased levels of vitamin C. In sum, vitamin C levels in seminal plasma were found to be positively correlated with normal sperm morphology and inversely correlated with subfertility [78,79]. Interestingly, vitamin C supplementation decreased sperm DNA damage and lipid peroxidation in men with low levels of vitamin C [80].

Vitamin E (α-tocopherol), a fat-soluble vitamin, is closely linked with vitamin C (that is water-soluble) and oxidative stress, and its impact on semen parameters is similar to the ones attributed to vitamin C. In fact, spermatozoa incubation with vitamin E showed a decrease in spermatozoa lipid peroxidation and higher motility after exogenous stress induction [81]. Moreover, one month of vitamin E treatment increased the fertilization rate in men with a low fertilization rate in IVF cycles [82].

l-carnitine and its derivatives (quaternary ammonium compounds) are also known for their important role in seminal plasma redox equilibrium. As seen with vitamins C and E, carnitine concentration on seminal plasma were positively correlated with sperm concentration, motility, and morphology in a study that compared l-carnitine in the seminal plasma of infertile men with fertile donors [83]. Moreover, l-carnitine and l-acetyl-carnitine treatment in asthenozoospermic men was found to increase sperm motility and free radical scavenging in the semen [83]. In infertile men with oligoasthenoteratozoospermia, l-carnitine and l-acetyl-carnitine supplementation increased all sperm parameters in these patients (concentration, motility, and morphology). Moreover, the most improvement was related to the individuals with more impaired sperm parameters at baseline [84].

Supplementation with trace elements, such as zinc (a powerful free radical scavenger), was also found to regulate asthenozoospermia by enhancing the capability of free radical scattering in the seminal plasma and decreasing apoptosis and DNA fragmentation in sperm cells by diminishing oxidative stress [85]. This is probably due to the capacity of zinc to neutralize superoxide ions [86]. Selenium is another trace element that is found to be decreased in non-normozoospermic men, when compared to the levels seen in fertile men [87]. The presence of this trace element, as well as zinc, in the seminal plasma was found to be positively correlated to total sperm motility, evidencing the role of trace elements in ROS neutralization for male fertility [88]. Additionally, the selenium binding protein 1 was found to be downregulated in men with low sperm motility [89]. Unfortunately, little is known about the role of this protein in selenium metabolism and bioavailability [90]. Selenium not only has direct antioxidant properties but also has been related to the activity of the antioxidant selenoprotein glutathione peroxidase (GPX) [91]. This enzyme is involved in the reduction of glutathione (γ-l-glutamyl-l-cysteinylglycine) that, like selenium, presents direct antioxidant capabilities and is involved in the activity of enzymatic antioxidants, as GPX and glutathione reductase (GR). Total glutathione concentration on the seminal plasma of non-normozoospermic men was found to be reduced in relation to the one of normozoospermic men [88]. Moreover, glutathione supplementation increased the sperm motility of infertile patients [92]. It is believed that glutathione has an especially important role in the regulation of the ROS created by mitochondria in the sperm midpiece [93]. However, this effect is mostly credited to the increased activity of GPX and GR rather than the direct antioxidant capability of glutathione. This subject will be further discussed in the next topic, where the role of protein antioxidants will be discussed.

All the studies described above show that the lack of one specific non-protein antioxidant increases the ROS content of the seminal plasma. However, the information available highlights that the general lack of antioxidants is the cause of the enhanced oxidative reductive potential, and not one specific antioxidant. The lack of sufficient antioxidant capacity of the seminal plasma can lead to an increase in oxidative damage of sperm cells, seen in the form of DNA fragmentation, lipid and protein oxidation, and decreased mobility, which ultimately led to sperm cell apoptosis and diminished motility. Although some non-protein antioxidants seem to have more antioxidant relevance (e.g., vitamin C) than others, we could not find any study that showed a compensation of other non-protein antioxidant to restore the seminal plasma antioxidant capability. This is probably because the concentrations of most of these non-protein antioxidants in the organism is diet-dependent. Thus, the organism cannot simply produce more non-protein antioxidants. However, this is not the case for protein antioxidants, whose expression and activity can be regulated by the organism. The expression and activity of antioxidant proteins in individuals with subfertility/infertility will be discussed in more depth in the next topic.

It is evident that diet has a great impact on the antioxidant capability of seminal plasma and hence in male fertility. Naturally occurring antioxidants, such as vitamin C, vitamin E, and trace elements such as zinc and selenium, are essential as the first line of defense against ROS [94], demonstrating the importance of proper free radical disposal for male fertility. Yet, these nutrients can be naturally acquired by a healthy diet. Thus, this reveals the importance of proper nutrition and its beneficial effect on male fertility potential. In fact, supplementation with the combination of the multiple antioxidants presented above was found to have an impact on repairing abnormal sperm parameters and increasing chances of conception [95,96,97].

### 3.2. Protein Antioxidants Significance for Male Fertility

In contrast to non-protein antioxidants, which are no longer effective after ROS neutralization [98], catalytic antioxidants are a renewable resource. The large majority of these enzymes are secreted into the seminal plasma by the epididymis and prostate [99].

**Figure 3:**
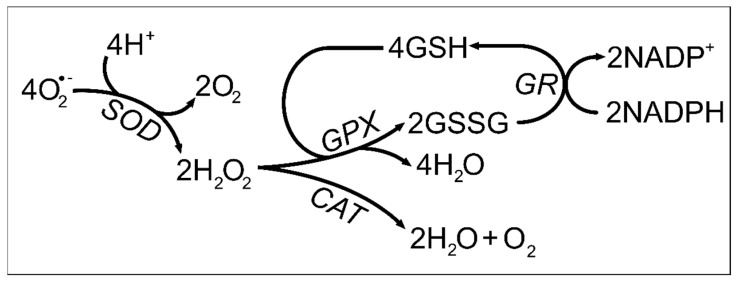
Role of catalase, superoxide dismutase, glutathione peroxidase, and glutathione reductase on oxidizing agent neutralization. O_2_—Superoxide anion; H^+^—Proton; O_2_—Oxygen; SOD-Superoxide Dismutase; H_2_O_2_—Hydrogen peroxide; H_2_O—Water; GPX—Glutathione Peroxidase; CAT—Catalase; GSH—Glutathione; GSSG—Glutathione disulfide; GR—Glutathione reductase; NADP^+^—Nicotinamide adenine dinucleotide phosphate; NADPH-Reduced nicotinamide adenine dinucleotide phosphate.

Antioxidant enzymes, such as catalase (CAT), superoxide dismutase (SOD), GPX, and GR, are known to be one important line of defense for inner-cell free radical protection. Since these proteins are also found in the seminal plasma, their prevalence could be connected to the fertility outcome. In brief, SOD scavenges superoxide anions and is responsible for its conversion to hydrogen peroxide (H_2_O_2_) and oxygen. Then, H_2_O_2_ can be degraded by CAT and catalyzed into water and oxygen [100], or cleared by GPX, which uses glutathione as an electron donor (Figure 3) [101].

In a study performed by Khosrowbeygi and collaborators, CAT activity was found to be decreased in seminal plasma of men with asthenozoospermia, asthenoteratozoospermia, and oligoasthenoteratozoospermia when compared to the values found in seminal plasma of normozoospermic men. On the other hand, no differences have been described in SOD between infertile/subfertile patients and fertile donors [61]. Moreover, the individuals with lower CAT activity were the ones with a decreased total antioxidant capacity, which can highlight the importance of this enzyme as an antioxidant threshold for seminal plasma. Another study reported that the seminal plasma of infertile men presented higher SOD activity, while the CAT activity was similar to that of seminal plasma of fertile men [102]. Yet, another study described that SOD and CAT activities were correlated with sperm concentration [103], both being enhanced in subfertile/infertile men. This highlights contradictory results between the studies published on the subject. This could be due to different comorbidities, from which oxidative stress increased and infertility evolved. As an example, metabolic diseases and cancers tend to increase ROS production and the proteins mentioned above tend to be upregulated [104]. Another reason for the disparity found in the literature is the study of different types of infertility origins in the same study. Two of the studies (both of which reported increased CAT activity) are focused on sperm parameters, while the one that did not find any difference in CAT activity did not study sperm parameters but only the inability to impregnate [56,98,99].

More recent studies took a proteomic analysis of this concept and were able to share some interesting results. Dias and collaborators compared the proteome profile of the seminal plasma of fertile men with high (ROS > 102.2 RLU/s/10^6^ sperm) and normal (ROS < 102.2 RLU/s/10^6^ sperm) levels of ROS [105]. This study identified upregulated expression of proteins involved in antioxidant capacity, such as haptoglobin (HP), peroxiredoxin 4 (PRDX4), and the protein S100A9, in men with high ROS content in the seminal plasma. HP is known to be important in protecting lipoproteins from oxidative damage by metal ions, such as copper and iron, present in hemoglobin [106,107]. PRDXs are known to be one of the spermatozoa first line of defense against oxidative stress [108] whereas S100A9 has a role in inflammatory regulation and bactericidal properties [109].

These antioxidant proteins could have been unaccounted for as agents in the maintenance of sperm function in seminal plasma with high ROS content. In another seminal plasma proteomic study, this time in obese men, the antioxidant proteins HP and S100A9 were also found to be upregulated when compared to the expression in men with normal body mass index (BMI) [110]. Generally, obese men have increased ROS concentration in the seminal plasma. This is usually caused by the continuous inflammatory state that these patients suffer, justified by the continuous formation of new adipose tissue [111]. Moreover, obese/overweight patients tend to have decreased levels of plasma antioxidants, such as vitamin C [112], probably due to an unbalanced diet. Thus, upregulated expression of these proteins in the seminal plasma could be an attempt of the body to decrease the high ROS levels and maintain proper sperm function. However, this was not sufficient since, in this study, obese men had decreased non-progressive motility, morphology, and increased sperm DNA fragmentation in relation to healthy men [110].

PRDXs are also known to have important roles in free radical homeostasis in sperm cells [113]. However, concerning its content on the seminal plasma of idiopathic infertile men and fertile donors, no difference was found both for PRDX1 and PRDX6 [114]. On the other hand, a more recent study described that seminal plasma content of PRDX5 was higher in asthenozoospermic in relation to the one of normozoospermic men [115]. Hence, PRDX5 could be the PRDX with more relevant antioxidant activity in the seminal plasma. PRDX5 is known to be localized in the cytosol, mitochondria, and peroxisomes of the cells and is not regarded as an extracellular PRDX [116]. Human sperm cells also express PRDX5 [116]. It would be interesting to understand if the increased PRDX5 is from apoptotic spermatozoa that had the PRDX5 upregulated due to oxidative stress or if epithelial cells of the epididymis can secrete more PRDX in individual with excessive ROS production with the goal of compensating with more antioxidant capacity. Taking into consideration that most studies report that men with impaired fertility have decreased total antioxidant capacity, it is noticeable that the majority of the literature reports also describe that non-protein antioxidants are decreased in men with fertility problems, whereas protein antioxidants, such as PRDX, HP, and SOD, are more expressed or/and active than those of men with proven fertility. Thus, the increased concentration of protein antioxidants can highlight the lack of non-protein antioxidants, which triggers an upregulation of proteins with antioxidant properties to regulate the levels of ROS. In fact, one protein with antioxidant capabilities that was found to be downregulated in men with impaired fertility was the extracellular GPX3. It was described that healthy donors had increased activity in GPX3, up to 10-fold that of men with idiopathic infertility [117]. Moreover, samples characterized with severe asthenozoospermia, oligozoospermia, and teratozoospermia were the ones with the lesser GPX activity in relation to the one measured in normozoospermic men [118]. Thus, GPX activity in the seminal plasma could be a biomarker of male fertility potential [117,118]. Most importantly, the dysregulation of the glutathione cycle could be the most crucial mechanism to oxidative stress regulation in the seminal plasma. In animals, female mice had significantly more cases of miscarriage when impregnated by *Gpx5-null* male mice as compared to those impregnated be wild-type mice. The authors hypothesized that this was probably due to the insufficient management of H_2_O_2_ and the subsequent increase in sperm cell DNA fragmentation [119], that leads to miscarriage. It is worth mentioning that this study was performed aiming at the GPX5 expression in sperm cells and not in the seminal plasma. However, it highlights the importance of glutathione peroxidase-like activity for proper sperm function. Glutathione cycle, performed by GPX and GR, is able to scavenge H_2_O_2_ and lipid peroxides that interrupts the chain reaction of lipid peroxidation [93].

Albumin is a protein that is greatly represented in seminal plasma. In the blood, albumin is able to bind to and neutralize ROS and oxidative agents [120]. In respect to male fertility, the first report relating albumin to sperm health stated that albumin was able to decrease medium oxidative stress after a cryopreservation/thawing cycle. The authors also hypothesized that albumin supplementation in samples from men with leukospermia could be a way to protect the sperm from oxidative stress for assisted reproductive techniques [121]. Follow-up studies reported that seminal albumin is positively correlated with sperm concentration and normal morphology in sperm cells. However, no correlation was found between albumin and sperm motility or DNA fragmentation [122]. This fact points to the possibility that the positive effects that albumin has on sperm are due to reasons other than ROS scavenging, since sperm motility and DNA integrity are two major markers of impaired antioxidant capability in the semen. The secondary role of albumin in regulating seminal ROS can be further explained by the fact that it only accounts for 10% of total seminal plasma proteins, which is considerably less than the percentage seen in the circulating serum [123]. Despite that, increasing albumin concentration through supplementation could be an interesting approach for ROS regulation after ejaculation of individuals with impaired sperm parameters due to oxidative stress.

The complex network of ROS management in a complex fluid such as the seminal plasma is a mechanism that still needs attention from the scientific community. The altered antioxidant protein expression and its relationship with the concentration of the different non-protein antioxidants and the contribution of each type of antioxidants to the total antioxidant capacity of the seminal plasma still have many secrets to be revealed.

## 4. A Brief Overview of the Physiological Roles of ROS in the Female Reproductive Tract

ROS are highly regulated at different levels, being a major player in multiple signaling pathways on multiple cellular functions. Indeed, multiple studies revealed the presence of ROS in ovaries [124], fallopian tubes [125], and embryos [126]. Normal ROS levels are important in regulating different signaling transduction pathways in folliculogenesis and corpus luteum oocyte maturation [127]. Additionally, ROS are closely related to the ovarian function and are involved in the regulation of ovarian steroid biosynthesis and secretion, primordial follicle recruitment, and ovulation [128,129]. It is also a key mediator in the fertilization process and post-fertilization events (egg implementation), and fetoplacental development [127,130,131] embryonic development disorders, and responsible for the regulation of both innate and acquired immune system in embryonic cells [132]. However, the ROS levels have to be closely regulated to ensure proper tissue function. One example of that is the regulation of antioxidant enzyme SOD activity during the menstruation process. In the genesis of menstruation and endometrial shedding, SOD activity decreases in the late secretory phase while ROS levels increase [133]. Additionally, all follicular stages present SOD expression, demonstrating that ROS management plays a role in oocyte maturation, folliculogenesis, ovarian steroidogeneses, and luteolysis [134]. Although the presence of ROS can help to trigger the mechanisms involved in fecundation, it can also exert negative aspects in mammalian ovaries. An imbalance between ROS overproduction and low antioxidant defense will lead to functional disorder. As in men, it is already described that oxidative stress is linked to reduced female reproductive capacity [134]. ROS can affect meiotic processes, spindle integrity, cytoskeleton structure, chromosomal aneuploidy, and even the acceleration of apoptosis [134]. It can also be associated with conditions such as polycystic ovary syndrome (PCOs), hydrosalpinges, endometriosis, and unexplained subfertility [135]. In fact, a large part of reproductive diseases is caused by inflammation, leading to changes that promote the body’s protection or, in worsening scenarios, cell death. Like in seminal plasma, macrophages, leucocytes, and cytokines are considered major sources of ROS in cycling ovaries [136]. Furthermore, pre-ovulatory luteinizing hormone (LH) was demonstrated to activate polymorphonuclear cells, thus amplifying the ovarian production of ROS [137]. In preovulatory follicles, excessive antioxidant impairs LH-stimulated progesterone secretion and ovulation-related gene expression [138]. Additionally, LH can bind to the ovarian surface, which can, consequently, stimulate the release of tumor necrosis factor-alpha (TNF-α). There is a close relationship between cytokines and antioxidant activity in the female reproductive tract. An example of that is the positive correlation between TNF-α with peritoneal fluid and endometriosis [139]. Thus, inflammation-resulting ROS are culprits of the rise of oxidative stress; however, other ROS sources can also have effects in human fertility. As an example, nitric oxide (NO) in the fallopian tubes has a crucial role in tubular contractility. Rosseli and collaborators disclosed that a deficiency of NO is responsible for a tubal motility dysfunction, culminating in retention of the ovum and delayed sperm transportation [140]. During the second trimester of normal pregnancy, oxidative stress is considered normal for proper gestation progress. High levels of NO are positively associated with multiple fertility outcomes, including the maturation of oocytes, ovulation, gamete transportation, sperm–oocyte interaction, and fertilization [141]. However, increased levels of NO in the fallopian tubes can be toxic to spermatozoa [140]. Superoxide anions are considered the major source of ROS in the placenta [142]. Moreover, it has been observed that women with early onset preeclampsia produce larger amounts of superoxide anions [143]. Additionally, these women have decreased total antioxidants status and placental GPX [144]. Furthermore, lack of vitamin C seems to be associated with an increased risk of preeclampsia, and some studies have shown that supplementation with multivitamins can lower the risk of preeclampsia in normal or underweight women [145]. In women with idiopathic infertility, a reduction in antioxidants, such as glutathione and vitamin E, and a higher concentration of ROS are observed [146]. Thus, an adequate antioxidant concentration is essential to protect the oocyte and the entire follicles from an excess of ROS and, consequently, from oxidative damage, to maintain a good quality of gametes and thus support reproduction [147]. However, the molecular mechanisms and roles have not been fully elucidated.

### The Importance of Female Reproductive Tract Protein and Non-Protein Antioxidants in Fertility and Embryo Development

Over the years, the function of ROS in female reproduction has been emphasized more than the effect of the antioxidants. Human oocytes have natural antioxidant defenses, such as classical enzymatic antioxidants (SOD, CAT, GPX) and glutathione [148]. However, the oocyte itself is not able to mobilize all the necessary antioxidant enzymes. The oocytes are protected against ROS, through the surrounding cumulus cells (COCs) [149]. These cells expressed multiple antioxidant enzymes. The follicular fluids surrounding the COCs act as an antioxidant buffer to keep the redox homeostasis. The physiological level of H_2_O_2_ in the follicular fluid may be a marker of healthy oocyte development [150]. During follicle growth, H_2_O_2_ promotes cell apoptosis, which is neutralized by glutathione and follicle-stimulating hormone (FSH) in healthy conditions [151]. Thus, a balance between ROS and antioxidants is necessary for a proper ovulation process. ROS produced in the preovulatory follicle is considered an important inductor of ovulation, where increased ROS concentration is observed before ovulation, whereas antioxidants inhibit the resumption of the first meiotic division [138]. On the other hand, antioxidants promote the progression of the second meiotic division. So, the process of oocyte maturation and meiosis I and II is mainly influenced by variable amounts of ROS and antioxidants [124]. This data demonstrates that the relationship between ROS and antioxidants is more complex than expected [148].

Antioxidants in the fallopian tube and uterus help to remove excess ROS [147] and thus provide the best environment for embryo development. The different antioxidants end up having different purposes, concerning the phase in which ovulation and/or pregnancy is. For instance, GPX can maintain low levels of hydroperoxidases inside the follicle and thus play an important role in gametogenesis and fertilization [152]. Glutathione and GPX are antioxidants responsible to neutralize the free radical and lipid peroxides to maintain the intracellular homeostasis and redox balance. Glutathione is considered the major representative of non-protein antioxidants present in oocytes and embryos [124]. At early stages, glutathione concentrations increase in oocytes after LH surge-induced ovulation. The glutathione depletion prevents against sperm chromatin decondensation and male pronucleus formation, indicating that glutathione is essential in fertilization events and embryonic development since it is responsible for the reduction in sperm nucleus disulfide bonds [153]. Gardiner and Reed compared fertilized mouse oocytes to non-fertilized and demonstrated that glutathione increases gamete viability, as well as the fertilization process, in fertilized mice [154]. Additionally, it is believed that glutathione concentration can be modulated by gonadotropin signaling during the preovulatory period. Indeed, FSH stimulation enhanced glutathione concentration [155]. GR, the enzyme responsible to convert the glutathione disulfide back into glutathione (Figure 3), can play a role in ovarian function, by keeping glutathione at reduced levels [156]. Furthermore, GPX has been reported to play significant roles in gametogenesis and IVF [152]. Curiously, this family of enzymes is present in higher amounts in patients who experience recurrent abortions [157].

Despite low information available, some studies demonstrate that CAT contributes to follicular development, the oestrous cycle, and steroidogenesis events in the ovaries [158]. Moreover, in obese and infertile women were observed higher levels of CAT activity, demonstrating that the follicular fluid of obese women was associated with a higher CAT activity indicating excessive oxidative stress [159]. Surprisingly, in the previous study, higher levels of SOD activity were not detected. SOD enzyme families are present in multiple tissues and ovaries [160]. It is responsible for the protection role in protecting the oocyte during their maturation phase by converting superoxide anions to hydrogen and water [161]. SOD1 is responsible to neutralize superoxide anions in the cytoplasm of oocytes [162], while SOD2 concentration is increased in the corpus luteum in order to eliminate the ROS produced in mitochondria by cytokine and inflammation [124]. Moreover, SOD3 is the only SOD isoform that is present in the zona pellucida [163]. Interestingly, high SOD activity in follicular fluid was correlated with lower fertilization rates in humans.Oocytes that did not become fertilized presented more SOD activity that those whose oocytes did become fertilized [164]. Matzuk and collaborators demonstrated that *Sod1*-null mice had a reduced fertility and *Sod2*-deficient mice die within three weeks of birth. Furthermore, the same authors completed transplantation of ovaries from the postnatal *Sod2*-deficient mice. These mice showed all stages of folliculogenesis, including corpora lutea, and gave rise to viable offspring [165].

As discussed, zinc is a key element for the proper functioning of the reproductive system, since the cells of this system differentiate and proliferate extensively, and these processes are zinc-dependent. It is necessary for spermatozoa development, ovulation, fertilization, normal pregnancy, fetal development, and parturition [166,167]. Zinc is one of the cofactors of antioxidant enzymes, such as glutathione, CAT, and GPX [168]. Diabetic women are more prone to develop PCOs [169], which is a syndrome linked to oxidative stress and infertility. Nowadays, there are medications available to lower high glucose levels. Zinc particles are one of the options available. l-carnitine, a small water-soluble molecule, exerts its antioxidant effects directly as a free radical scavenger or indirectly by affecting antioxidant enzymes [170]. It can also, protect mitochondria against oxidative stress, and reduces apoptosis. The combination of l-carnitine with zinc nanoparticles as a supplement or drug may improve the fertility process in diabetic patients. Majidi and collaborators performed a study in female rats, where female Wistar rats received a single intraperitoneal injection of streptozotocin. After 21 days of treatment, a co-treatment with l-carnitine (200 mg/kg/day) and Zinc nanoparticles (10 mg/kg/day) reduced stress oxidative markers (malondialdehyde and carbonyl protein) and increased glutathione levels, as well as CAT and SOD activities in ovarian tissue, when compared to the diabetic group, where rats received an intraperitoneal injection of streptozotocin. It also increased serum levels of FSH, LH, estradiol, and progesterone. In women with PCOs, zinc supplementation was able to show a positive effect by increasing the total antioxidant capacity, with a simultaneous decrease in protein carboxyl and malondialdehyde concentrations [171].

Antioxidants required during oocyte maturation, fertilization, and early embryonic development, as well as ROS proportion, fluctuate at different stages. The potential effects of antioxidants in in vitro maturation are considered a scientific challenge since it is reliable to understand the exact amount of pro-oxidants and antioxidants needed to improve the quality of in vitro matured human oocytes. Ali and his collaborators performed a study where they evaluated the non-protein (cysteine and *N-*acetyl cysteine) and enzymatic antioxidant (SOD and CAT) supplements in bovine oocytes. The addition of antioxidants during in vitro maturation revealed a significantly impaired development of bovine embryos [172]. Levine and Morita, in 1985, described that vitamin C deficiency can cause atrophy of ovarian follicular atresia and premature resumption of meiosis and so is one cause of infertility [173].

Supplementary antioxidants, such as vitamin D and the combination of pentoxifylline and vitamin E, have multiple benefits for female fertility, which includes improved blood circulation in the endometrium, decreased insulin resistance, lowered hyperandrogenism, fertile cervical mucus, and an influence on prostaglandin synthesis and steroidogenesis [174,175]. Some studies previously demonstrated that taking a multivitamin tablet, which includes acid folic and vitamins C, D, and E, may increase fertility. A cohort study demonstrated that antioxidants (different combinations of l-arginine, vitamin E, myo-inositol, d-chiro-inositol, carnitine, selenium, vitamin B complex, vitamin C, vitamin D + calcium, Coenzyme Q10, and omega-3 polyunsaturated fatty acids) may improve clinical pregnancy rates when compared with a placebo or no treatment [176]. This suggested that, among subfertile women with an expected clinical pregnancy rate of 19%, the rate among women using antioxidants would be 25 and 30%. However, the heterogeneity of the study was high, since the trials englobed women with different indications and antioxidants tested. Nevertheless, 28 trials demonstrated a contradictory suspicion, where the use of antioxidants had no difference in rates of multiple pregnancies or rates of ectopic pregnancy [176]. Furthermore, five randomized controlled trials demonstrated that the supplementation of Coenzyme Q10 increased clinical pregnancy when compared with placebos or with no treatment. Additionally, no difference was observed in groups of live births and miscarriage rates [177].

*N*-acetyl-cysteine has antioxidant properties whose main function is to increase intracellular glutathione concentration and/or directly scavenge free radicals [178,179]. The combination of *N*-acetyl-cysteine and vitamin B9 (folic acid), revealed promising results since pregnancy loss was improved in patients with unexplained recurrent pregnancy losses [180]. Melatonin (*N*-acetyl-5-methoxy tryptamine), a hormone associated with control of the sleep–wake cycle, also has a role in reproduction, especially in the human preovulatory follicle [181]. Melatonin and its metabolites are direct free radical scavengers and modulate gene transcription for antioxidant enzymes [182]. It is undeniable that oxidative stress is one of the biggest obstacles to overcome. Indeed, women who failed to become pregnant through IVF–embryo techniques were provided with 3 mg of melatonin *per day*, 600 mg of vitamin E *per day*, or both from the fifth day of the previous menstrual cycle to the day of oocyte retrieval. After administration of these compounds, women revealed a lower concentration of DNA oxidated [183].

However, there is little high-quality information evidence to show that taking antioxidants will provide any benefit for infertile couples (Table 2). To overcome fertility problems, many couples undergo ovarian stimulation, as well as different assisted reproductive technologies (ART), and compared to these expensive and invasive techniques, antioxidants are widely available and are cheaper. Nonetheless, their true meaning on subfertility is still unclear [184].

## 5. Role of Seminal Plasma on Fertility Techniques and Importance of Its Antioxidants in the Female Reproductive Tract

The impact of the usage of seminal plasma on artificial reproductive techniques are starting to be studied in multiple species, including humans. For example, in cattle, the infusion of seminal plasma into the reproductive tracts of Holstein cattle increased the birth weight of heifer calves born after insemination with X-sorted semen but did not alter pregnancy or live birth statistics after insemination [186]. Seminal plasma also has an important role in the conception of embryos by IVF at the time of transfer into the female tract. In mice, embryo transfer protocols usually employ recipients exposed to seminal plasma by mating with vasectomized males. Fetal loss and other issues are greater when embryos are transferred after pseudopregnancy achieved with no exposure to seminal plasma [187]. In humans, a randomized trial in couples undergoing ART techniques has shown a significant improvement in embryo transfer after exposure to seminal plasma [188]. Hart and Norman demonstrated a higher proportion of transferred embryos in 6–8 weeks in women that were exposed to semen around the time of embryo transfer compared to those who abstained [189]. Some meta-analyses provided additional information about seminal plasma in clinical pregnancy rates. The meta-analysis conducted by Crawford and collaborators [190] demonstrated a significant improvement in clinical pregnancy rates, approximately 23%, while Saccone and collaborators demonstrate a 20% improvement [191]. However, other authors’ analysis concluded that seminal plasma may effectively increase clinical pregnancy rates, but the finding should be regarded with caution since there is not enough evidence to determine the impact on live births [192]. These data demonstrate that further studies are warranted to better quantify the impact of seminal plasma on live birth after ART techniques and thus find the difference between coitus and exogenous delivery of seminal plasma.

Recent studies disclosed that conception in the absence of seminal plasma in mice impairs embryo development and changes fetal development. Bromfield and collaborators demonstrated that conception without seminal plasma in mice with seminal vesicle deficiencies (surgically rendered) (SVX) lead to a reduction in fecundity and changed fetal and neonatal outcomes. Females that mated with these males had a reduction in progression of pregnancy and fewer implanted embryos compared to intact mated females. Subsequently, embryo transfer experiments demonstrated that seminal plasma has also a role in reprogramming phenotype. An increase in adiposity was observed in adult male progeny when normal 2-cell embryos were transferred to females mated with SVX males [193]. Furthermore, in pigs, Álvares-Rodriguez and collaborators evaluated whether pathways are induced by the deposition of seminal plasma in porcine female internal genital tracts. They concluded that the semen, after either mating or artificial insemination, presented a higher expression of genes associated with sperm transport and binding and oxidative stress, such as phosphatidylinoditol 3-kinase and protein Kinase B (PI3k-Akt), forkhead box O (FoxO) signaling, and others. They also showed that seminal plasma itself can alter the transcriptome and consequently be a key player on the transport and binding of spermatozoa–oocyte [194]. Moreover, a subset of miRNAs acquired during epididymal transit is related to the modification of other female tract signaling pathways, such as transforming growth factor beta (TGF-β) [195]. This is further proof that the female immune environment is influenced by sperm-borne miRNAs, in addition to paternal programming and influencing embryonic development [196,197]. Seminal plasma has a wide variety of extracellular vesicles [198]. In humans, the majority of exosomes are prostasomes. They transport proteins, nucleic acid, lipids, and prostate-specific proteins [199,200]. Regarding fertility, prostasomes can deliver agents that alter sperm function during ejaculation [201]. They bind to capacitated sperm at a neutral or slightly alkaline pH [202] and then fuse at the slightly acidic pH [203], the same as it is in the female tract environment. Additionally, exosomes isolated from pigs were revealed to elicit gene expression changes in endometrial cells in the female human tract [204]. Given these properties, studies of the composition of seminal plasma, particularly this type of exosomes, can help to unravel the mechanisms by which seminal plasma mediates paternal effects on offspring health.

In human beings, the direct effect of seminal plasma signaling activity on long-term health is not clear. There are clinical observations that demonstrated the possible effects of seminal plasma in the female reproductive system, including in specific diseases. The impact of seminal plasma factors on human immunodeficiency viruses (HIV) infections in vivo has been investigated [205]. Sperm can assist in the efficient transmission of HIV [206] through the interaction of HIV and sperm heparin sulfate. However, other factors seemed to inhibit HIV infectivity; Mucin-6 and seminal plasma cationic peptides are responsible for this [207].

Seminal plasma does not only support the sperm cell during its journey through the male reproductive tract but also has crucial roles in the female reproductive tract. After ejaculation, the interaction between the semen and the vaginal epithelium provokes multiple adaptations of the female reproductive tract [208]. Some seminal plasma constituents will trigger regulation of endometrial tissue and remodulation of uterine receptivity, whereas the sperm cells also trigger an immune response [209,210]. This immune reaction on the part of the female is normal as a mechanism to protect them from foreign organisms. In fact, the connection between spermatozoa and the female tract is important for the normal influx of leucocytes [211]. This early leukocyte attack allows the female reproductive system to eliminate spermatozoa with poor fertilization capacity based on poor DNA integrity [212]. Thus, only the most capable sperm have the ability to escape these harsh conditions, which will result in a sperm selection suited for healthy fertilization [193]. In fact, only approximately 0.004% of all motile ejaculated sperm can reach the oviduct, demonstrating the meticulous selection performed by the female reproductive tract [213]. However, without the protection of seminal plasma and, to a lesser extent, the vaginal reproductive tract fluid antioxidants this number could be much lower. As mentioned, sperm cells are particularly vulnerable to oxidative stress and are dependent of the elements of seminal plasma and of both vaginal and uterine fluids to survive the female immune response. Upon semen reception, vaginal fluid will increase it levels of inflammatory response, antimicrobial peptides, medium acidification, and ROS as a consequence of the interaction between semen and the vaginal epithelium [214]. Seminal plasma is able to protect the sperm cells from most of these adverse effects by being constituted by high concentration of buffers, antimicrobial minerals and proteins, and antioxidants [214]. Moreover, seminal plasma has anti-inflammatory proteins that are able to minimize the immune response of the female by interacting with the vaginal, cervical, and uterine epithelium [215], which will indirectly control the ROS formation of the immune response. An example of such protein is adrenomedullin (ADM), which is secreted by the prostate, and its level in the seminal is correlated with sperm motility [216]. Additionally, seminal fluid ADM may play an anti-oxidative role in the female reproductive tract [217]. Liao and collaborators demonstrate that, in rats [217], ADM can protect the sperm from the oxidative damage caused by ROS, generated by leukocytes and dead spermatozoa in the ejaculate [218] or by the uterus [219]. ADM substantially decreased the level of different cytokines in the uterine cavity in the first hours after ejaculation [217]. Seminal plasma is also rich in TGF-β isoforms that act as an immunosuppressor. This role is very important in the male reproductive tract and protects sperm from seminal leucocytes [220], yet it can also be detrimental for inhibiting the female immune response directed at the sperm cells [215].

Despite all these immune suppressors, some excessive ROS formation is unavoidable. Some originate in the female medium (mainly by leukocytes in the cervix and vaginal microbiome) and others are believed to be formed by the sperm cells through their long journey to the oocyte [212]. The management of the latter was already discussed above. However, excessive female ROS formation could be an additional challenge from which the seminal plasma must protect the sperm cells. One example of an oxidative molecule present in the female reproductive tract that was found to have deleterious effect on sperm is the NO. Seminal plasma NO concentration is positively correlated to male infertility [221]. This particular ROS is important for the contraction of the uterine and oviduct cavities that also assist in sperm motility for oocyte fertilization [222]. However, this NO can also affect sperm. Interestingly, seminal plasma has an inhibitory role on the protein NO synthase activity in brain tissue [223]. One could assume that a similar process can be happening in the female reproductive tract for sperm protection against oxidative damage; however, this is only a hypothesis and tests should be made to confirm it.

Seminal plasma is also responsible for the protection of sperm cells against the vaginal microbiome. Recent studies show that interaction between the vaginal microbiome and sperm cells can decrease sperm cell motility, which can inhibit fertilization [224]. Healthy seminal plasma is rich in zinc [85], which is known to have antibacterial properties capable of protecting sperm cells from microbial organisms in the male reproductive system [225]. A similar protective role of zinc can occur in the female reproductive system by controlling the vaginal microbiome and not allowing the loss of sperm function. Some seminal plasma proteins also have antibacterial activities, such as semenogelins (SEMG). Homologues SEMG1 and SEMG2 are the most represented proteins in semen [226]. These proteins interact with prostate-specific antigens, causing their degradation [227]. Interestingly, the SEMG-derived peptides presented bactericidal properties in the presence of zinc [225]. During ejaculation, and after the combination of the prostate and the seminal vesicles secretion (where the prostate-specific antigen and SEMG are present, respectively), the SEMG will start to be degraded. At the female tract, SEMG-derived peptides should be able to assist in sperm protection from damage derived from the vaginal microbiome.

Other seminal plasma constituents are able to bind to the post-acrosomal region of the sperm head and go along with the sperm to downstream parts of the female reproductive tract. There, seminal plasma is already scarce after dilution throughout the female reproductive tract medium; however, sperm seems to be able to bind some proteins and other molecules that will have important roles in future events [215]. Heparin is an example of a molecule that binds to proteins bound to sperm cells, and evidence suggests that this molecule has crucial roles in sperm capacitation [228,229]. Despite some contradictory results on heparin ability to directly neutralize ROS [230,231] its presence has been related to extracellular SOD activity [232]. In the oviduct, as an example, seminal plasma SOD activity probably does not present much antioxidant effect to be enhanced by heparin; however, SOD is also present in the lumen medium of the female reproductive tract [160]. This symbiotic-like effect between female SOD and male sperm cells was confirmed in a canine study [233]. As far as we know, no study has investigated the effect of heparin bound to sperm on medium SOD activity; this would be an interesting mechanism to further research. Notwithstanding, evidence suggests that heparin concentrations in seminal plasma are directly correlated with sperm parameters, particularly to sperm concentration and motility [229]. The authors of this study justified that fact by highlighting that the majority of seminal plasma proteins can bound heparin. Thus, less heparin can only indicate less seminal plasma proteins in general, which can have important roles on sperm maturation [229]. Moreover, after seminal plasma dilution, the oviductal epithelium secretes proteins to the lumen fluid. These cells were reported to secret proteins that protect the sperm from oxidative damage and increase the activity of antioxidant enzymes, such as SOD and GPX, in sperm cells [234]. This strengthens the theory that sperm cells can increase their antioxidant defenses in oxidative environments due to exogenous stimulation, and this helps to maintain sperm cell protection from oxidative stress for the last stages of their journey. At that moment, hyperactivation takes place, which is characterized by enhanced mitochondrial function and thus enhanced ROS production [235]. Taking this into consideration, the sperm protection supplied by the oviductal epithelium protein secretion should be crucial for sperm to reach the oocyte and resist the novel ROS formation. In a study performed with bovine sperm, oviductal GPX, SOD, and CAT were found to be important for the sperm protection against oxidative damage, which can, ultimately, decrease sperm viability and motility [185].

**Figure 4:**
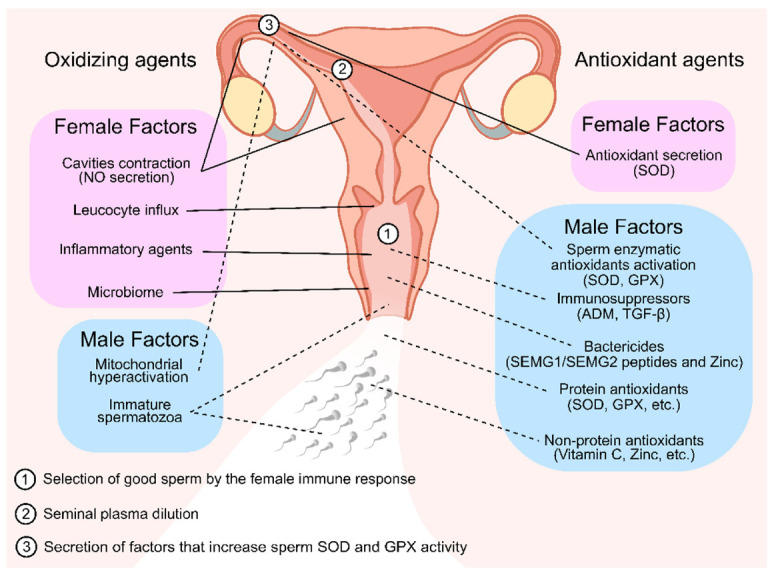
Oxidizing and antioxidant agents of seminal plasma and female reproductive tract fluid that are linked with selecting or protecting the sperm cells throughout their journey to fertilization. In the first stages of the sperm journey, they encounter multiple oxidizing agents in the vaginal fluid. To counter that, many antioxidizing agents are present in the seminal plasma. Both these facts contributed for the selection and protection of good sperm for fertilization (1). In later stages, after seminal plasma dilution (2), seminal plasma antioxidant protection is scarce; thus, it is possible that the oviductal epithelium secretes a factor that stimulates the sperm antioxidant defenses by increasing SOD and GPX activity (3) as well and secreting antioxidant enzymes to the lumen, which are necessary to protect the sperm from the endogenous ROS formation in the hyperactivation stage. Solid lines represent female factors. Dashed lines represent male factors. NO—Nitric Oxide; SOD—Superoxide Dismutase; GPX—Glutathione Peroxidase; SEMG—Semenogelins.

Some examples of the importance of the reproductive fluid antioxidants to human fertility are described above and summarized at Figure 4; however, many other mechanisms are surely yet to be revealed. The study of the interactions between seminal plasma and the female reproductive fluid antioxidants still presents many obstacles, since it is challenging to study this process in vivo and almost impossible to replicate the condition in in vitro experiments. Despite not being able to fully understand yet the specific contribution that a good spermatozoon has on a damaged female reproductive tract through ROS or vice versa, it is undeniable that seminal plasma has multiple positive aspects in female reproductive stress to ensure proper fertilization and hence embryo development. Thus, understanding the modification of the female genital profile triggered by seminal components that interact with the internal genital lining during the ovulatory period may contribute to improving success rates and outcomes in fertility.

## 6. Conclusions

The study of the male and female reproductive tract fluids is a valuable tool to access fertility issues, with particular interest when no other infertility cause is apparent. As shown above, many reports present evidence that seminal plasma antioxidants can have an important role in the protection of the sperm cells against endogenous ROS production and from male and female immune responses, as well as helping sperm cells achieve capacitation. It is worth mentioning that, as many of the studies are performed in animal models, its applicability in humans is yet to be fully understood. Another interesting hypothesis worth further investigation is the possibility of female reproductive tract antioxidants assisting the sperm cells with extra antioxidant capabilities after seminal plasma dilution and during embryo development. If so, it would be fair to consider a complementary protection supplied by both reproductive fluids in sperm protection to ensure fertilization. Thus, dysregulation of the total antioxidant capacity of these fluids has a deleterious effect on fertility. The study of reproductive fluids should be an alternative and a complement to fertility evaluation; however, their importance is far to reunite a consensus regarding the applied methods to evaluate the antioxidants and to establish cut-off levels for fertility issues. Additionally, is important to consider that the sources of antioxidants in seminal plasma/ejaculate can be multiple and difficult to identify, so in this way, it is not possible to establish a pathological condition for a lack of antioxidants in this fluid. Moreover, understanding the modification of the female genital profile triggered by seminal components that interact with the internal genital lining during the ovulatory period may contribute to improving success rates and outcomes in fertility. A deeper comprehension of the mechanisms that are behind, as well as the effective role of the antioxidants, more particularly, SOD, CAT, and GPX activity, and how to manage them in reproduction could be a step ahead for the unexplained infertility couple.

## Figures and Tables

**Table 1:** Information available on the principal seminal plasma antioxidants and the main male reproductive glands responsible for its secretion.

Reproductive Glands	Antioxidants	References
Epididymis	Carnitines	[44]
Seminal Vesicle	Vitamin C	[44]
Prostate	Zinc	[45,46,47,48]
	Selenium	
	Glutathione peroxidase	
	Glutathione reductase	
	Superoxide dismutase	
	Catalase	
	Albumin	

Note: The glands presented are regarded as the major contributor of the antioxidants and cannot be the exclusive secretor.

**Table 2:** Main antioxidant nutrients and enzymes present in male and female reproductive tract fluids and its relationship with fertility parameters. Arrows illustrate the levels of the nutrients/enzymes in the conditions presented. ↓ decreased and ↑ increased.

	Male	Female	References
Vitamin C	↓ Asthenozoopermia	↓ Early-onset preemclampsia ↓ Atrophy of ovarian follicular atresia	[78,79,143,173]
Vitamin E	↓ Lipid peroxidation ↓ Asthenozoopermia	↓ Idiopathic infertility	[81,82,146]
	↑ Associated with increased fertility rate		[82]
Cartinine	↑ Good Sperm parameters	↑ Fertile diabetic patients	[83,84,170]
Trace elements (e.g., Zinc)	↓ DNA Fragmentation ↓Asthenozoopermia		[85,86]
Glutathione Peroxidase	↓Asthenozoospermia, oligozoospermia, and teratozoospermia	Important role in gametogenesis and fertilization	[92,117,152]
	Crucial for oocyte encounter		[185]
Glutathione	Prevents against sperm decondensation and male pronucleus formation	Present in oocytes and embryo	[124,153]
Catalase	↓ Asthenozoospermia, asthenoteratozoospermia, and oligoasthenoteratozoospermia	↑ in obese women	[61,159]
SOD	↑ in infertile/subfertlie patients ↑ Sperm concentration	Fluctuates during menstruation process ↓ low fertilization rates	[102,103,133,164]
